# High-Frequency Workpiece Image Recognition Model Integrating Multi-Level Network Structure

**DOI:** 10.3390/s24061982

**Published:** 2024-03-20

**Authors:** Yang Ou, Chenglong Sun, Rong Yuan, Jianqiao Luo

**Affiliations:** 1 School of Mechanical Engineering, Chengdu University, Chengdu 610106, China; ouyang@my.swjtu.edu.cn (Y.O.); yuanrong27@126.com (R.Y.); 2 School of Mechanical Engineering, Southwest Jiaotong University, Chengdu 610031, China; sunclwlkq@163.com; 3 College of Electrical and Information Engineering, Hunan University, Changsha 410082, China

**Keywords:** network structure, hybrid attention, image recognition, deep learning

## Abstract

High-frequency workpieces have the characteristics of complex intra-class textures and small differences between classes, leading to the problem of low recognition rates when existing models are applied to the recognition of high-frequency workpiece images. We propose in this paper a novel high-frequency workpiece image recognition model that uses EfficientNet-B1 as the basic network and integrates multi-level network structures, designated as ML-EfficientNet-B1. Specifically, a lightweight mixed attention module is first introduced to extract global workpiece image features with strong illumination robustness, and the global recognition results are obtained through the backbone network. Then, the weakly supervised area detection module is used to locate the locally important areas of the workpiece and is introduced into the branch network to obtain local recognition results. Finally, the global and local recognition results are combined in the branch fusion module to achieve the final recognition of high-frequency workpiece images. Experimental results show that compared with various image recognition models, the proposed ML-EfficientNet-B1 model has stronger adaptability to illumination changes, significantly improves the performance of high-frequency workpiece recognition, and the recognition accuracy reaches 98.3%.

## 1. Introduction

With the advancement of science and technology, the global manufacturing industry is developing rapidly towards high quality, high efficiency, and high intelligence [1,2,3]. High-frequency workpieces are one of the most important components in aerospace equipment. The quality, timeliness, and intelligence of their processing are all important factors affecting the development of the aerospace industry. During the processing of the workpiece, the workpiece is bound to the pallet with an RFID tag, and the processing content to be completed in the process is automatically loaded through scanning by the RFID tag reading and writing device. However, during the heat treatment stage of the workpiece, the separation of the workpiece from the pallet causes the failure of the original radio frequency chip of the workpiece, which affects the automation and intelligence of subsequent processing. Therefore, image recognition technology is used to identify the workpiece image after heat treatment and the workpiece image before heat treatment and then associate the processing content corresponding to the image number again and attach a new RFID tag to the high-frequency workpiece after heat treatment. The new RFID tag is the same as the tag before heat treatment. Radiofrequency chips with the same content are used to complete the intelligent process of subsequent finishing and other processing procedures. In order to improve the intelligence level of high-frequency workpiece processing, image recognition technology can be introduced into the processing process. The image recognition results are associated with the drawing number of the currently processed high-frequency workpiece, and the next step of processing can be automatically loaded through the manufacturing execution system (MES). At present, image recognition of high-frequency workpieces faces the following challenges: (1) The same types of workpieces have complex internal textures. (2) Different types of workpieces have small differences in characteristics. (3) The quality of the workpiece image is greatly affected by changes in acquisition posture and lighting.

Image recognition technology has been widely used in different fields, and many researchers have proposed a variety of recognition algorithms [4,5,6,7,8,9,10,11,12,13,14,15,16,17,18,19]. In [13], the author built a five-layer memristor-based CNN to perform MNIST10 image recognition and achieved a high accuracy of more than 96%. In [15], the author proposed a fastener classification model, which can divide fasteners into four types, including normal, partially worn, missing, and covered. In the industrial field, researchers have proposed some effective algorithms for image recognition of mechanical parts. It can be divided into traditional algorithms based on mathematical models and deep learning algorithms based on convolutional neural networks. In the first category, Xu et al. proposed a template matching algorithm RTMM using ring feature templates to solve the problem of low accuracy of traditional matching methods caused by the presence of different sides of parts in the image [20]. Yin et al. proposed a fast positioning and recognition algorithm based on equal-area ring segmentation to solve the problem of complex textures and high similarity after precision machining of high-frequency components, which makes it difficult to distinguish [21]. Wang et al. proposed a method of part sorting based on fast template matching, which accelerates the process of part recognition by improving the template matching method. Furthermore, the efficiency of the part sorting system based on machine vision is improved [22]. In the second category, Yang et al. proposed a joint loss-supervised deep learning recognition algorithm. This algorithm first builds an image feature vector encoding model based on a convolutional neural network, uses angle margin loss to replace SoftMax loss to reduce the distance between features within the workpiece class, and then introduces isolation loss to increase the distance between features of heterogeneous workpieces [23]. Zhang et al., proposed a multi-branch feature fusion convolutional neural network (MFF-CNN) to solve the difficult problems of multi-surface distribution of features and light sensitivity when automatically classifying automobile engine main bearing cover parts [24]. In addition, researchers have proposed improved Inception V3 [25] and Xception [26] for the identification of threaded connector parts.

The above algorithm can overcome the impact of illumination changes on recognition results to a certain extent; however, its research objective is relatively simple and cannot be effectively applied to the recognition of high-frequency workpieces that are complex within classes, have small gaps between classes, and have changeable postures. In order to effectively solve the problem of low recognition rate of high-frequency workpieces with the characteristics of complex intra-class and small gaps between classes under complex illumination changes, we propose in this paper a novel high-frequency workpiece image recognition model that uses EfficientNet-B1 [27] as the basic network and integrates multi-level network structures. The main contributions in this paper are threefold.

(1) We introduce a lightweight mixed attention module (LMAM) to extract global workpiece image features with strong illumination robustness, and the global recognition results are obtained through the backbone network.
(2) We use a weakly supervised area detection module to locate the locally important areas of the workpiece, which is then introduced into the branch network to obtain local recognition results.
(3) We combine the global and local recognition results in the branch fusion module to achieve the final recognition of high-frequency workpiece images.

Experimental results on a high-frequency workpiece dataset made in the laboratory show that compared with various image recognition algorithms, the proposed algorithm has stronger adaptability to illumination changes and significantly improves the accuracy of high-frequency workpiece recognition.

The remainder of this paper is organized as follows: Section 2 outlines our method. Section 3 presents the experimental result of a high-frequency workpiece dataset made in the laboratory. Section 4 concludes this paper.

## 2. The Proposed Model

### 2.1. Overall Framework

**Figure 1:**
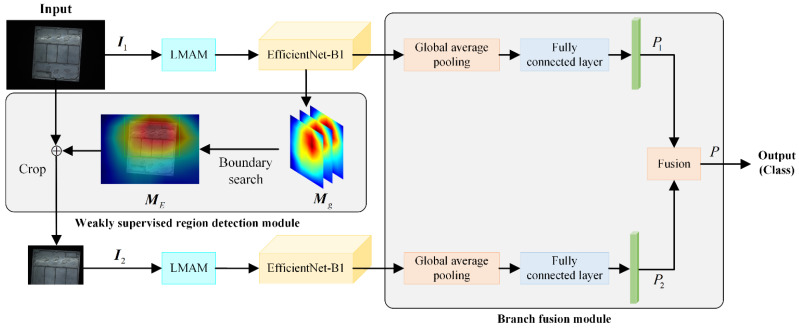
The framework of the proposed model.

The framework of the high-frequency workpiece recognition model proposed in this paper that integrates global attention and local attention is shown in Figure 1. It mainly consists of three modules: lightweight mixed attention module (LMAM), weakly supervised region detection module, and branch fusion module. First, the whole workpiece image ***I***_1_ is input into the lightweight mixed attention module (LMAM) to obtain enhanced features, and then, the multi-layer feature map ***M***_g_ and recognition results *P*_1_ are generated through the lightweight network EfficientNet-B1. Second, in the weakly supervised area detection module, the local workpiece image ***I***_2_ is intercepted based on the whole workpiece image ***I***_1_ and multi-layer feature map ***M***_g_, and it is imported into the LMAM and EfficientNet-B1 of another branch to obtain the recognition result *P*_2_. Finally, the branch fusion module is used to fuse the results of the two branches to obtain the final recognition result *P*.

### 2.2. The Lightweight Mixed Attention Module (LMAM)

In actual industrial production environments, the surface of a high-frequency workpiece is easily affected by factors such as uneven illumination and a large change in illumination, resulting in spots, shadows, insufficient light, and other phenomena in the collected workpiece images. If low-quality workpiece images are directly input into the network to extract features, effective image features cannot be obtained, and it is difficult to accurately identify the categories of high-frequency workpieces. In order to overcome the impact of the above interference information on workpiece recognition, this paper uses the LMAM module to enhance the features of the workpiece image, as shown in Figure 1.

**Figure 2:**
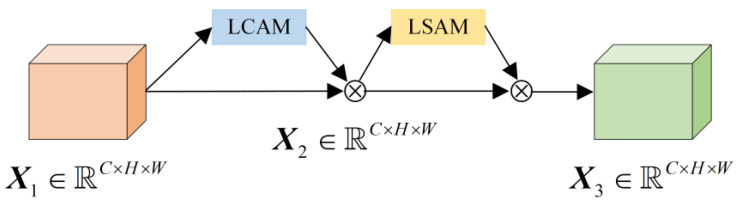
Structure diagram of LMAM.

Inspired by the convolutional block attention module (CBAM) [28] and efficient channel attention (ECA) [29], this paper proposes the LMAM shown in Figure 2 for preliminary screening. It uses the lightweight channel attention module (LCAM) and the lightweight spatial attention module (LSAM) to replace the channel attention module (CAM) and spatial attention module (SAM) in CBAM, respectively.

#### 2.2.1. The Lightweight Channel Attention Module (LCAM)

**Figure 3:**
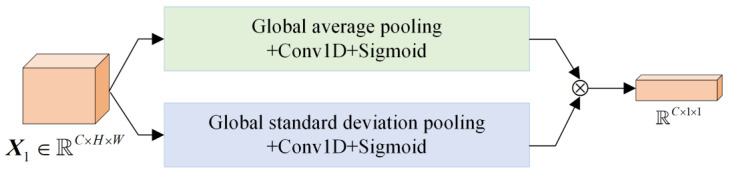
Structure diagram of LCAM.

Since the maximum pooling in CAM loses too much information, the LCAM in this article uses global standard deviation pooling to replace the global maximum pooling. Then, one-dimensional convolution allows the network to focus on the learning of effective channels with less calculation, and its structure is shown in Figure 3.

Using global average pooling and global standard deviation pooling on the input feature map $X_{1}\in {\mathbb{R}}^{C\times H\times W}$, the channel information description maps are obtained using the following two formulas, respectively.

(1) $$z^{m}=\frac{1}{H\times W}\sum_{h=1}^{H}\sum_{w=1}^{W}X_{1}\left(h,w\right)$$

(2) $$z^{sd}=\sqrt{\frac{1}{H\times W}\sum_{h=1}^{H}\sum_{w=1}^{W}{\left(X_{1}\left(h,w\right)-z^{m}\right)}^{2}}$$

where *h* and *w* are the coordinates in the height and width directions, respectively. *H* and *W* are the height and width of the image, respectively.

***z****^m^* and ***z****^sd^* accumulate global information in different ways and then perform one-dimensional convolution on them, respectively. Note that ***z****^m^* can effectively extract salient information, and ***z****^sd^* can extract differential information. After a combination of convolution and activation, the channel attention weight is obtained.

(3) $$M_{c}=\sigma \left(F_{m}^{k}\left(z^{m}\right)\right)⊗\sigma \left(F_{sd}^{k}\left(z^{sd}\right)\right)$$

where $F_{m}^{k}\left(\cdot \right)$ and $F_{sd}^{k}\left(\cdot \right)$ are one-dimensional convolution, respectively. k is the convolution kernel size. $\sigma \left(\cdot \right)$ is sigmoid function.

The convolution kernel size k can be adaptively calculated by the formula in the literature [18].

(4) $$k=\psi \left(C\right)={\left|\frac{\log_{2}\left(C\right)+b}{\gamma }\right|}_{odd}$$

where C is the number of channels, γ and b are set to 2 and 1, respectively. odd represents the nearest odd number.

#### 2.2.2. The Lightweight Spatial Attention Module (LSAM)

**Figure 4:**
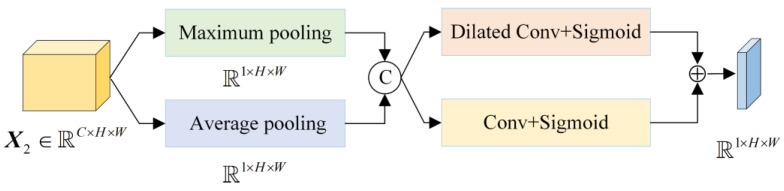
Structure diagram of LSAM.

The LSAM proposed in this paper is shown in Figure 4. In view of the small target difference between classes in the high-frequency workpiece dataset, a convolution kernel of size 3 × 3 is used to replace the convolution kernel of size 7 × 7 in SAM. At the same time, in order to obtain multi-scale context information, a dilated convolution with a kernel size of 3 × 3 is added in parallel. Since dilated convolution will produce a grid effect, different receptive fields are summed and combined. There are only 18 parameters in the entire convolution process, which greatly reduces the number of parameters compared with the 49 parameters of SAM.

Perform maximum pooling and average pooling on each pixel of the input feature map $X_{2}\in {\mathbb{R}}^{C\times H\times W}$ by channel, then perform channel concatenation on the two obtained feature maps. Next, perform convolution with a kernel size of 3 × 3 and a dilated convolution with a kernel size of 3 × 3, where the dilation rate is set to 1 and 2, respectively. In order to ensure that the size of the feature map remains unchanged, the padding parameters need to be set, and, finally, the spatial attention weight is obtained through activation and addition operations.

(5) $$M_{s}\left(X_{2}\right)=\sigma \left(F_{C}^{3\times 3}\left(\left[P_{\max}\left(X_{2}\right);P_{avg}\left(X_{2}\right)\right]\right)\right)⊕\sigma \left(F_{D}^{3\times 3}\left(\left[P_{\max}\left(X_{2}\right);P_{avg}\left(X_{2}\right)\right]\right)\right)$$

where $F_{C}^{3\times 3}\left(\cdot \right)$ and $F_{D}^{3\times 3}\left(\cdot \right)$ denote the convolution and the dilated convolution is a kernel size of 3 × 3, respectively. $P_{\max}\left(\cdot \right)$ and $P_{avg}\left(\cdot \right)$ denote the maximum pooling and average pooling, respectively.

### 2.3. The Weakly Supervised Region Detection Module

High-frequency workpieces have the characteristics of many types and small differences between classes, and the small differences between workpieces often appear in specific local areas. Based on these characteristics, this paper proposes to use a weakly supervised region detection module, which includes two mechanisms, boundary search and cropping, to locate areas with significant differences in the workpiece image, as shown in Figure 1.

The global multi-layer feature map $M_{g}$ of the input image is generated through the backbone network EfficientNet-B1, and the energy map $M_{E}$ is obtained by superimposing the feature maps of all channels. In order to eliminate the interference of negative elements, all elements of $M_{E}$ are normalized to $\left[0,1\right]$ to obtain the scaled energy map.

(6) $${\hat{M}}_{E}\left(i\right)=\frac{M_{E}\left(i\right)-\min\left(M_{E}\right)}{\max\left(M_{E}\right)-\min\left(M_{E}\right)}$$

where $\max\left(M_{E}\right)$ and $\min\left(M_{E}\right)$ denote the maximum value and the minimum value in $M_{E}\left(i\right)$, respectively.

In order to further improve the positioning accuracy, bilinear interpolation is used to upsample ${\hat{M}}_{E}$ to the size of 25 × 25.

(7) $${\bar{M}}_{E}=bilinear\left({\hat{M}}_{E}\right)$$

Then, ${\bar{M}}_{E}$ is aggregated into two one-dimensional structured energy vectors.

(8) $$\left\{\begin{array}{c} V_{w}=\sum_{i=0}^{H}{\bar{M}}_{E}\left(i,W\right)\\ V_{h}=\sum_{j=0}^{W}{\bar{M}}_{E}\left(H,j\right) \end{array}\right.$$

where $V_{w}$ and $V_{h}$ denote one-dimensional structured energy vectors along the width and height directions of space, respectively.

Taking $V_{w}$ as an example, extract the energy of different elements.

(9) $$\left\{\begin{array}{c} E\left[0:W\right]=\sum_{i=0}^{W}V\left(i\right)\\ E\left[x_{1}:x_{2}\right]=\sum_{i=x_{1}}^{x_{2}}V\left(i\right) \end{array}\right.$$

where $E\left[0:W\right]$ denotes the energy sum of all elements in the width vector, and $E\left[x_{1}:x_{2}\right]$ denotes the energy of the area with width from $x_{1}$ to $x_{2}$.

Define the key area in the global image as occupying the smallest area and meeting the following condition:

(10) $$\left\{\begin{array}{c} E\left[x_{1}:x_{2}\right]/E\left[0:W\right]>\gamma \\ E\left[y_{1}:y_{2}\right]/E\left[0:H\right]>\gamma \end{array}\right.$$

where γ denotes the preset threshold.

The width boundary coordinate $\left[x_{1}:x_{2}\right]$ and the height boundary coordinate $\left[y_{1}:y_{2}\right]$ of the area can be found automatically by using the boundary search mechanism. Then, a cropping mechanism is used to intercept effective workpiece information and valuable background information from the original image according to the entire boundary coordinate $\left[x_{1}:x_{2},y_{1}:y_{2}\right]$, and the local workpiece image $I_{2}$ is finally obtained.

### 2.4. The Branch Fusion Module

In order to simultaneously utilize the global information and local information of the image and weigh the role of the two types of information in workpiece image recognition, a branch fusion module is proposed to take into account the recognition results of the two branches to further improve the accuracy of the recognition, as shown in Figure 1. It should be noted that in the model proposed in this paper, the two channel attention modules do not share parameters to extract workpiece features at different scales. The calculation formula of the fused recognition score is as follows:

(11) $$P=\mu P_{1}+\left(1-\mu \right)P_{2}$$

where $P_{1}$ and $P_{2}$ denote the recognition result of the global image and the recognition result of the local image, respectively. And μ denotes the balancing factor that weighs the recognition results of different branches.

## 3. Experimental Results

### 3.1. Dataset

**Figure 5:**
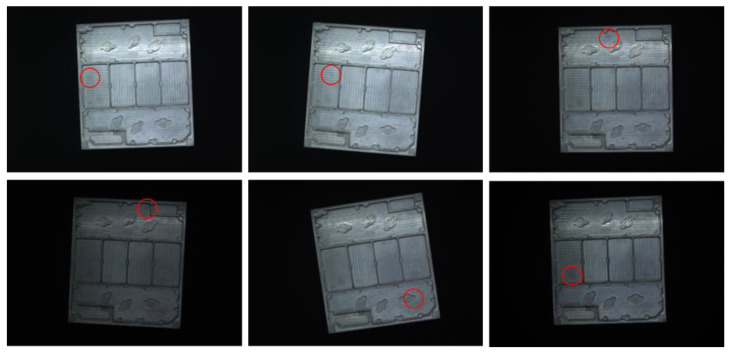
Some high-frequency workpiece image samples.

The high-frequency workpiece image dataset used in the experiment comes from a research institute in China. The dataset contains 20 different categories of workpieces. Each category of workpieces has 1000 images, totaling 20,000 images. The size of the image is 3822 × 2702. The dataset is randomly divided into a training set and a validation set in a ratio of 7:3. Figure 5 shows examples of six different categories of high-frequency workpieces, where the red circle indicates the small differences between the six categories. It can be seen from Figure 5 that for each type of high-frequency workpiece image, its internal texture is characterized by complex texture; for different types of high-frequency workpiece images, the differences between the classes are small. In addition, there are also local bright spots caused by uneven lighting in the image. Therefore, classifying multi-category high-frequency workpiece images is an equally challenging task.

### 3.2. Experimental Settings

The training process of ML-Efficient-B1 is carried out by the Adam optimizer [30], where the initial learning rate is set to $1\times 10^{-4}$. The batch size and image size are set to 8 and 224 × 224, respectively. The training process is performed until 50 epochs using a machine with NVIDIA GeForce GTX 1660 SUPER GPU (NVIDIA is located in Santa Clara, California, USA). The implementation is carried out using the PyTorch1.8 package [31]. Note that the weight parameter initialization of the network is obtained by using pre-training on ImageNet.

### 3.3. Model Parameter Selection

The cropping range threshold γ determines the size of the effective area extracted from the global image, further affecting the accuracy of workpiece recognition. If γ is too small, excessive loss of workpiece features will result. On the contrary, if γ is too large, the network will not be able to focus on important local features. Therefore, the intercepted area should be limited to a reasonable range. This article limits it to $\left[0.60,0.80\right]$. In addition, the parameter μ in Equation (11) also greatly affects the network’s emphasis on different branches.

In order to determine the optimal threshold, the balance factor was first set to 0.6, and then the recognition accuracy of high-frequency workpieces was tested when the values of γ were 0.6, 0.65, 0.70, 0.75, and 0.80, respectively. The experimental results are shown in Table 1.

As can be seen from Table 1, as the cropping range threshold increases, the accuracy first increases and then decreases. When the threshold is 0.70, the algorithm in this paper achieves the best performance. Therefore, 0.70 is selected as the final threshold.

In order to evaluate the impact of the balance factor μ on the recognition results, first γ is fixed at 0.70, and then the high-frequency workpiece recognition accuracy is tested when μ takes different values. The experimental results are shown in Table 2.

As can be seen from Table 2, when μ is 0.6, the high-frequency workpiece image obtains the highest recognition accuracy. As μ decreases, the recognition accuracy of high-frequency artifacts gradually decreases. This is because the cropped partial image contains less information, and relying too much on this part will cause the network to be unable to obtain better results. Therefore, we take μ=0.6 as the balance parameter.

### 3.4. Comparison of Recognition Performance

In order to verify the effectiveness of the high-frequency workpiece image recognition model proposed in this paper, a comparative experiment was conducted with a variety of image recognition algorithms. Comparison algorithms include RTMM [20], JLS-DL [23], EfficientNet [27], a mechanical parts identification algorithm based on convolutional neural network [32] (denoted as WorkNet-2), main bearing cover parts identification based on deep learning [24] (denoted as MFF-CNN), the part recognition algorithm based on improved convolutional neural network [33] (denoted as Xception-P), NOAH [34], and RAFIC [35]. The experimental results are shown in Table 3.

It is shown in Table 3 that the recognition performance of the proposed ML-EfficientNet-B1 for high-frequency workpiece images is significantly better than other comparison methods. Specifically, the increases over that of the EfficientNet, WorkNet-2, MFF-CNN, Xception-P, RTMM, JLD-DL, NOAH, and RAFIC methods on the high-frequency workpiece image dataset are 12.1%, 8.3%, 7.4%, 6.2%, 4.7%, 3.4%, 2.2%, and 1.0%, respectively.

**Figure 6:**
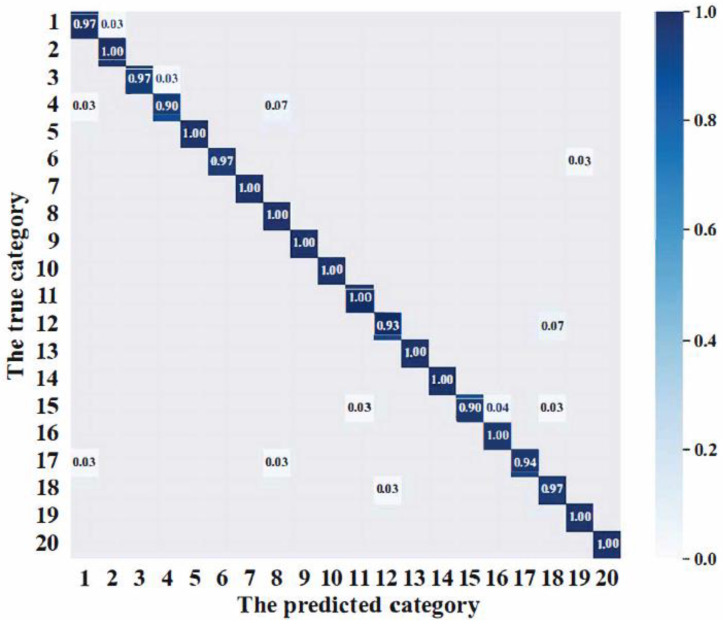
Confusion matrix on the high-frequency workpiece image dataset.

In addition, Figure 6 shows the confusion matrix of the recognition results of the proposed model. As can be seen from Figure 6, the recognition accuracy of the ML-EfficientNet-B1 for each type of high-frequency workpiece is above 90%.

### 3.5. Ablation Study

In this sub-section, we carry out an ablation study of the proposed ML-EfficientNet-B1 in order to show the new modules on improving the network performance. Specifically, based on retaining the EfficientNet-B1 network, the effect of the lightweight mixed attention module (LMAM), the weakly supervised region detection module (WSRDM), and the branch fusion module (BFM) on the entire model is verified by controlling variables. The experimental results are shown in Table 4.

The following can be seen from Table 4: (1) When directly using the EfficientNet-B1 network to classify high-frequency workpiece images, the recognition accuracy is 86.2%. (2) After adding the LMAM module based on the backbone network EfficientNet-B1, the accuracy rate is increased by 10.5%. This shows that the LMAM module has the ability to perceive feature information of different color channels, overcome the impact of illumination changes, and more effectively extract the features of high-frequency workpiece images. (3) On the basis of using the LMAM module, with further added WSRDM, the accuracy rate further increased by 0.7%, which shows that the feature learning of the WSRDM focuses on differentiated effective areas, which can improve the accuracy of recognition. (4) When LMAM, WSRDM, and WSRDM are used simultaneously, the accuracy rate is further improved by 0.9%, indicating that combining global information and local information can improve the recognition performance of high-frequency workpieces.

### 3.6. Visualization Results

**Figure 7:**
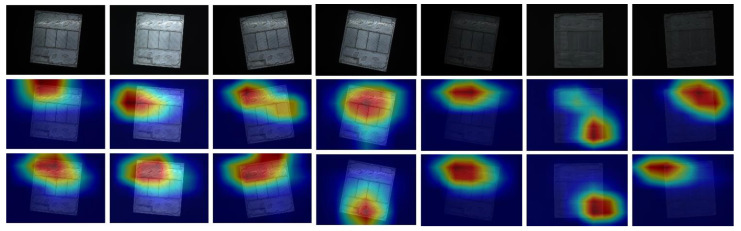
Attention feature visualization. Note that the first row denotes the original images, the second row denotes the results obtained by EfficientNet, and the third row denotes the results obtained by the proposed ML-EfficientNet-B1.

In order to verify the effectiveness of the proposed algorithm, some images are randomly selected for visual display on the same test set. Figure 7 shows the subtle differences between different categories of high-frequency workpieces and the visualization of attention features before and after algorithm improvement.

As can be seen from Figure 7, compared with the original EfficientNet network, the improved ML-EfficientNet-B1 model makes the network more focused on the area where the boss is located. Therefore, the proposed EfficientNet-B1 model can extract more discriminative workpiece image features, significantly improving the recognition performance of the network.

Table 5 demonstrates the inference time of the proposed ML-EfficientNet-B1 and the other algorithms on a machine with NVIDIA GeForce RTX 3090 GPU, for an image with the spatial resolution of 224 × 224. As seen from this table, the inference time of the proposed network is 0.133 (s), which is acceptable for providing a high performance.

### 3.7. Discussion and Limitation

In summary, the improved model proposed in this paper can effectively improve the recognition accuracy of multi-category high-frequency workpiece images, effectively solve the impact of illumination changes on high-frequency workpiece recognition results, and make full use of the global and local features of workpiece images. When the model in this paper is used to classify high-frequency workpiece images, it only uses the top surface image and does not consider the thickness information of the workpiece. It cannot be applied to the classification of high-frequency workpiece images with different thicknesses but the same top surface information. In subsequent algorithms, combining multi-view images can be considered to improve classification accuracy and the number of categories.

## 4. Conclusions

This paper proposes a high-frequency workpiece image recognition model that integrates multi-level network structures. First, LMAM is introduced to enhance the feature extraction capability of the network and reduce the impact of illumination changes on high-frequency artifact recognition results. Then, a weakly supervised area detection module is used to search and locate differentiated local images. Finally, the branch fusion module is used to effectively balance the network’s ability to capture global features and local features of the workpiece images. Experimental results show that compared with the original EfficientNet network, the model in this paper improves the recognition accuracy of high-frequency artifacts by 12.1%. In addition, compared with various image recognition methods, the model in this paper has a certain degree of improvement in the recognition accuracy of high-frequency workpieces. In the future, in order to better arrange it on the production line, we will study a more lightweight high-frequency workpiece classification model.

## Figures and Tables

**Table 1:** The impact of the size of threshold γ on the recognition results.

The Values of γ	Accuracy (%)
0.60	96.5
0.65	97.7
0.70	98.3
0.75	97.5
0.80	96.8

**Table 2:** The impact of balancing factors on recognition results.

The Values of μ	Accuracy (%)
0.0	96.7
0.1	97.2
0.2	97.4
0.3	97.6
0.4	97.7
0.5	97.8
0.6	98.3
0.7	97.5
0.8	97.0
0.9	96.3
1.0	96.1

**Table 3:** Recognition results of different algorithms.

Method	The Total Number of Images	The Number of Correct Recognitions	Accuracy (%)
EfficientNet [27]	6000	5142	86.2
WorkNet-2 [32]	6000	5370	90.0
MFF-CNN [24]	6000	5424	90.9
Xception-P [33]	6000	5496	92.1
RTMM [20]	6000	5586	93.6
JLS-DL [23]	6000	5664	94.9
NOAH [34]	6000	5763	96.1
RAFIC [35]	6000	5829	97.3
ML-EfficientNet-B1 (Our model)	6000	5868	98.3

**Table 4:** The impact of different modules on network performance.

LMAM	WSRDM	BFM	Accuracy (%)
×	×	×	86.2
√	×	×	96.7
√	√	×	97.4
√	√	√	98.3

× means that the module is not used; √ means that the module is used.

**Table 5:** Complexity of different algorithms.

Method	Execution Time
EfficientNet [27]	0.082 (s)
WorkNet-2 [32]	0.051 (s)
MFF-CNN [24]	0.104 (s)
Xception-P [33]	1.320 (s)
RTMM [20]	30.517 (s)
JLS-DL [23]	0.208 (s)
NOAH [34]	0.164 (s)
RAFIC [35]	0.185 (s)
ML-EfficientNet-B1 (Our model)	0.135 (s)

## Data Availability

The dataset that was generated and analyzed during this study is available from the corresponding author upon reasonable request, but restrictions apply to data reproducibility and commercially confident details.

## References

[1] Wübbeke J., Meissner M., Zenglein M.J., Ives J., Conrad B. (2016). Made in China 2025. Mercat. Inst. China Studies. Pap. China.

[2] Zenglein M.J., Holzmann A. (2018). Evolving made in China 2025. MERICS Pap. China.

[3] Zhou J. (2015). Intelligent Manufacturing—Main Direction of “Made in China 2025”. China Mech. Eng..

[4] Zhao H., Jia J., Koltun V. Exploring self-attention for image recognition. Proceedings of the IEEE/CVF Conference on Computer Vision and Pattern Recognition.

[5] Sampurno R.M., Liu Z., Abeyrathna R.M., Ahamed T. (2024). Intrarow Uncut Weed Detection Using You-Only-Look-Once Instance Segmentation for Orchard Plantations. Sensors.

[6] Alam L., Kehtarnavaz N. (2024). Improving Recognition of Defective Epoxy Images in Integrated Circuit Manufacturing by Data Augmentation. Sensors.

[7] Sheykhmousa M., Mahdianpari M., Ghanbari H., Ghamisi P., Homayouni S. (2020). Support vector machine versus random forest for remote sensing image classification: A meta-analysis and systematic review. IEEE J. Sel. Top. Appl. Earth Obs. Remote Sens..

[8] Zhao C., Qin Y., Zhang B. (2023). Adversarially Learning Occlusions by Backpropagation for Face Recognition. Sensors.

[9] Wang H., Ma L. (2023). Image Generation and Recognition Technology Based on Attention Residual GAN. IEEE Access.

[10] Yi X., Qian C., Wu P., Maponde B.T., Jiang T., Ge W. (2023). Research on Fine-Grained Image Recognition of Birds Based on Improved YOLOv5. Sensors.

[11] Li Z., Tang H., Peng Z., Qi G., Tang J. (2023). Knowledge-guided semantic transfer network for few-shot image recognition. IEEE Trans. Neural Netw. Learn. Syst..

[12] Xu C., Gao W., Li T., Bai N., Li G., Zhang Y. (2023). Teacher-student collaborative knowledge distillation for image classification. Appl. Intell..

[13] Yao P., Wu H., Gao B., Tang J., Zhang Q., Zhang W., Yang J.J., Qian H. (2020). Fully hardware-implemented memristor convolutional neural network. Nature.

[14] Zhou G., Li J., Song Q., Wang L., Ren Z., Sun B., Hu X., Wang W., Xu G., Chen X. (2023). Full hardware implementation of neuromorphic visual system based on multimodal optoelectronic resistive memory arrays for versatile image processing. Nat. Commun..

[15] Ou Y., Luo J., Li B., He B. (2019). A classification model of railway fasteners based on computer vision. Neural Comput. Appl..

[16] Luo J., He B., Ou Y., Li B., Wang K. (2021). Topic-based label distribution learning to exploit label ambiguity for scene classification. Neural Comput. Appl..

[17] Luo J., Wang Y., Ou Y., He B., Li B. (2021). Neighbour-based label distribution learning to model label ambiguity for aerial scene classification. Remote Sens..

[18] Xu Y., Feng K., Yan X., Yan R., Ni Q., Sun B., Lei Z., Zhang Y., Liu Z. (2023). CFCNN: A novel convolutional fusion framework for collaborative fault identification of rotating machinery. Inf. Fusion.

[19] Xu Y., Yan X., Sun B., Feng K., Kou L., Chen Y., Li Y., Chen H., Tian E., Ni Q. (2023). Online Knowledge Distillation Based Multiscale Threshold Denoising Networks for Fault Diagnosis of Transmission Systems. IEEE Trans. Transp. Electrif..

[20] Xu W., Li B., Ou Y., Luo J. (2021). Recognition algorithm for metal parts based on ring template matching. Transducer Microsyst. Technol..

[21] Yin K., Ou Y., Li B., Lin D. (2022). Fast identification algorithm of high frequency components based on ring segmentation. Mach. Des. Manuf..

[22] Wang Y., Chen H., Zhao K., Zhao P. A mechanical part sorting method based on fast template matching. Proceedings of the 2018 IEEE International Conference on Mechatronics, Robotics and Automation (ICMRA).

[23] Yang T., Ou Y., Su X., Wu X., Li B. (2023). High frequency workpiece deep learning recognition algorithm based on joint loss supervision. Mach. Build. Autom..

[24] Zhang P., Shi Z., Li X., Ouyang X. (2021). Classification algorithm of main bearing cap based on deep learning. J. Graph..

[25] Szegedy C., Vanhoucke V., Loffe S., Shlens J., Wojna Z. Rethinking the inception architecture for computer vision. Proceedings of the IEEE/CVF Conference on Computer Vision and Pattern Recognition (CVPR).

[26] Chollet F. Xception: Deep learning with depthwise separable convolutions. Proceedings of the IEEE/CVF Conference on Computer Vision and Pattern Recognition (CVPR).

[27] Tan M., Le Q. Efficientnet: Rethinking model scaling for convolutional neural networks. Proceedings of the International Conference on Machine Learning (ICML).

[28] Woo S., Park J., Lee J.Y., Kweon I. Cbam: Convolutional block attention module. Proceedings of the European Conference on Computer Vision (ECCV).

[29] Wang Q., Wu B., Zhu P., Li P., Zuo W., Hu Q. ECA-Net: Efficient channel attention for deep convolutional neural networks. Proceedings of the IEEE/CVF Conference on Computer Vision and Pattern Recognition (CVPR).

[30] Kingma D.P., Ba J.L. (2014). Adam: A method for stochastic optimization. arXiv.

[31] Paszke A., Gross S., Massa F., Lerer A., Bradbury J., Chanan G., Killeen T., Lin Z., Gimelshein N., Antiga L. (2019). Pytorch: An imperative style, high-performance deep learning library. Adv. Neural Inf. Process. Syst..

[32] Duan S., Yin C., Liu M. (2019). Recognition Algorithm Based on Convolution Neural Network for the Mechanical Parts. Adv. Manuf. Autom. VIII.

[33] Yang L., Gan Z., Li Y. (2022). Part recognition based on improved convolution neural network. Instrum. Tech. Sens..

[34] Li C., Zhou A., Yao A. (2024). NOAH: Learning Pairwise Object Category Attentions for Image Classification. arXiv.

[35] Lin H., Miao L., Ziai A. (2023). RAFIC: Retrieval-Augmented Few-shot Image Classification. arXiv.

